# A longitudinal survey of African swine fever in Uganda reveals high apparent disease incidence rates in domestic pigs, but absence of detectable persistent virus infections in blood and serum

**DOI:** 10.1186/s12917-015-0426-5

**Published:** 2015-05-13

**Authors:** Denis Muhangi, Charles Masembe, Ulf Emanuelson, Sofia Boqvist, Lawrence Mayega, Rose Okurut Ademun, Richard P Bishop, Michael Ocaido, Mikael Berg, Karl Ståhl

**Affiliations:** Department of Wildlife and Aquatic Resources, College of Veterinary Medicine, Animal Resources and Biosecurity, Makerere University, P. O. Box 7062, Kampala, Uganda; Department of Biological Sciences, College of Natural Sciences, Makerere University, P. O. Box 7062, Kampala, Uganda; Department of Clinical Sciences, Swedish University of Agricultural Sciences, P. O. Box 7054, SE-750 07 Uppsala, Sweden; Department of Biomedical Sciences and Veterinary Public Health, Swedish University of Agricultural Sciences, P. O. Box 7028, SE-750 07 Uppsala, Sweden; District Veterinary Office, under the Ministry of Agriculture, Animal Industry and Fisheries, Masaka, Uganda; Ministry of Agriculture, Animal Industry and Fisheries, P. O. Box 102, Entebbe, Uganda; International Livestock Research Institute (ILRI), P.O. Box 30709, GPO 00100 Nairobi, Kenya; Department of Disease Control and Epidemiology, National Veterinary Institute (SVA), SE-751 89 Uppsala, Sweden

**Keywords:** African swine fever (ASF), Epidemiology, Incidence rate, Risk factors, Smallholder farmers

## Abstract

**Background:**

African swine fever (ASF) is a fatal, haemorrhagic disease of domestic pigs, that poses a serious threat to pig farmers and is currently endemic in domestic pigs in most of sub-Saharan Africa. To obtain insight into the factors related to ASF outbreaks at the farm-level, a longitudinal study was performed in one of the major pig producing areas in central Uganda. Potential risk factors associated with outbreaks of ASF were investigated including the possible presence of apparently healthy ASF-virus (ASFV) infected pigs, which could act as long-term carriers of the virus. Blood and serum were sampled from 715 pigs (241 farms) and 649 pigs (233 farms) to investigate presence of ASFV and antibodies, during the periods of June-October 2010 and March-June 2011, respectively. To determine the potential contribution of different risks to ASF spread, a questionnaire-based survey was administered to farmers to assess the association between ASF outbreaks during the study period and the risk factors.

**Results:**

Fifty-one (21 %) and 13 (5.6 %) farms reported an ASF outbreak on their farms in the previous one to two years and during the study period, respectively. The incidence rate for ASF prior to the study period was estimated at 14.1 per 100 pig farm-years and 5.6 per 100 pig farm-years during the study. Three pigs tested positive for ASFV using real-time PCR, but none tested positive for ASFV specific antibodies using two different commercial ELISA tests.

**Conclusions:**

There was no evidence for existence of pigs that were long-term carriers for the virus based on the analysis of blood and serum as there were no seropositive pigs and the only three ASFV DNA positive pigs were acutely infected and were linked to outbreaks reported by farmers during the study. Potential ASF risk factors were present on both small and medium-scale pig farms, although small scale farms exhibited a higher proportion with multiple potential risk factors (like borrowing boars for sows mating, buying replacement from neighboring farms without ascertaining health status, etc) and did not implement any biosecurity measures. However, no risk factors were significantly associated with ASF reports during the study.

## Background

ASF is a fatal, haemorrhagic, viral infection of pigs caused by ASFV, an *Asfivirus* and the only member of the family *Asfarviridae*, which poses a threat to both commercial and smallholder pig farmers. It is currently endemic in at least 26 countries in sub-Saharan Africa [1] as well as on the Island of Sardinia (Italy), the Caucasus, parts of Russia, and in eastern part of the European Union where it was introduced in 2014. The disease can have a severe socio-economic impact on people’s livelihoods, food security and both regional and international trade [2].

In sub-Saharan Africa (SSA), the importance of pig production to food security and household incomes is growing and the numbers of pigs on the continent have increased almost threefold during the last decades [1, 3, 4]. This is as a result of a steady increase in demand for animal protein by a growing middle-class. Since most of the increase is taking place in smallholder or backyard husbandry systems with low levels of biosecurity, the growth of the sector creates disease prevention and control challenges [5]. A larger and denser pig population on the continent coupled with an increase in movements of pigs and pig products, as well as people, is most likely the main factor responsible for the upsurge of ASF in many new areas in SSA [1, 5]. The current ASF situation, with the rising numbers of endemically infected countries in SSA and Europe and more ASFV circulating globally constitutes a serious threat to ASF-free countries in Europe as well as Asia [6]. Further spill-over events from either Africa, the Caucasus or eastern Europe, as a result of increased movement of people and pig products, could lead to huge losses in international trade [5].

To date, three cycles involved in the transmission of ASF have been identified: the sylvatic cycle involving circulation of the virus between warthogs (*Phacochoerus africanus*) and soft ticks of the genus *Ornithodoros*, the tick-to-domestic pig cycle and lastly the domestic pig-to-pig cycle [7]. The sylvatic cycle is present in eastern and southern Africa and here, historically, it is considered the main source of outbreaks of ASF in domestic pigs [8]. Today, however, the disease has become endemic in the growing domestic pig populations in several countries in the region, including Uganda, with outbreaks mainly associated with movements of pigs and pig products [1]. A sufficiently large population provides a constant supply of naïve pigs, and is, in the low biosecurity setting dominating in SSA, therefore likely to allow maintenance of ASF without involvement of the sylvatic host. The presence of apparently healthy long-term carriers possibly shedding the virus as suggested by some authors [9–12] would further facilitate the indefinite perpetuation of the disease.

The aim of the study was to investigate the factors related to ASF outbreaks at farm-level, and maintenance of the disease in the domestic pig population, including the existence and possible role of apparently healthy ASFV infected pigs, which could act as long-term carriers of the virus.

## Results

### Farms sampled

A total of 715 pigs (241 farms) and 649 pigs (233 farms) were sampled at the first and second sampling time points, respectively. Despite one of the study’s conditions being that the farmers were to keep pigs until the next visit for follow up, eight of the farmers were not available at the second sampling point. Of these, two had sold off all pigs as a result of suspected ASF on the farm. The rest either sold them off for reasons other than ASF or they were not available for interview at the second visit.

**Fig. 1:**
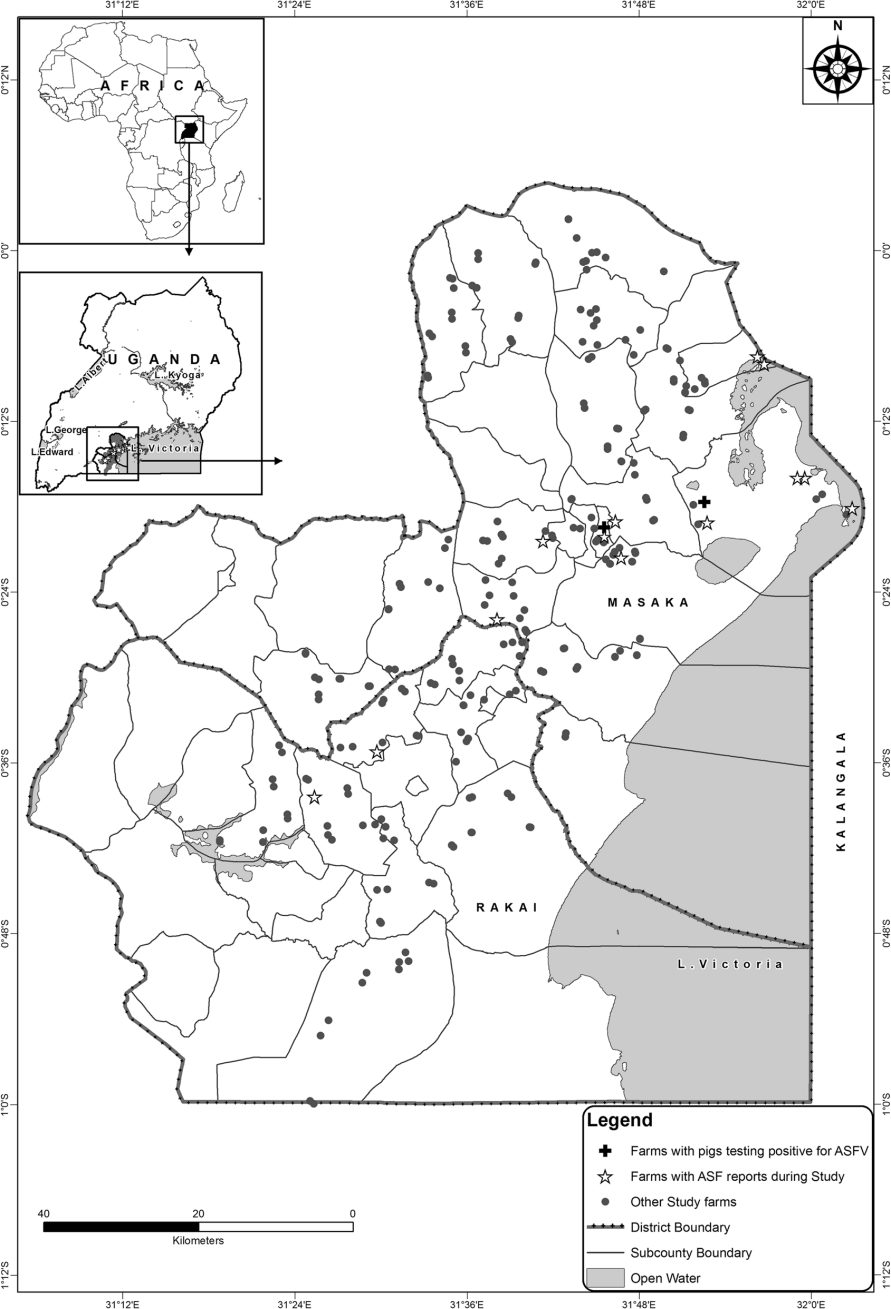
Distribution of pig farms reporting ASF in Masaka and Rakai, Uganda. Map of the study area showing the pig farms sampled (n = 241) in Masaka and Rakai, Uganda (2010–2011). The highlighted farms (star symbol) are the farms where farmers reported ASF during the study and cross symbol are those confirmed positive by RT-PCR

Four hundred seventy six (476) pigs on 161 farms from Masaka and 239 pigs on 80 farms were sampled from Rakai during the first sampling point (Fig. 1). Four hundred twenty two (422) pigs on 154 farms and 227 pigs on 79 farms from Masaka and Rakai, respectively were sampled during the second phase of the study (Fig. 1).

### Awareness and knowledge of ASF

The vast majority of the farmers were aware of ASF and could correctly describe two to three of the most important clinical signs. ASF was generally described as a disease that has no cure or vaccine, and that kills large numbers of pigs fast. Pigs develop high fever, lose appetite and die within two days. Discoloration of the skin (turning blue or red) is also mentioned.

### Incidence of ASF

A total of 51 (21 %) farms reported having had incidences of ASF one to two years preceding the first visit. Between the first and second visits, 13 (5.6 %) farms reported having experienced an outbreak of ASF (Fig. 1), with mortalities between 12–100 % (median 66 %). In one of the farms, ASF was confirmed as positive by RT-PCR in two out of the three pigs sampled. The incidence rates for ASF were estimated at 14.1 per 100 pig farm-years (95 % CI 7.7;23.5) and 5.6 per 100 pig farm-years (95 % CI 2.2;13.1) for the periods prior to and between the two visits, respectively. The difference in estimated incidence rates for the two periods was not statistically significant (*P*-value = 0.10).

### ASFV DNA and antibody detection

Genomic DNA was successfully extracted from all the pooled samples. All the pigs in the initial round tested negative for ASFV using RT-PCR. In the second sampling round, three pigs from two different farms tested positive (Ct values: 21.2, 37.9 and 38.8) (Fig. 1). These two farms were both located in areas where outbreaks of ASF had occurred. The corresponding true prevalences were estimated at 0 % (95 % CI 0; 0.6) and 0.5 % (95 % CI 0.1; 1.5) at the first and the second sampling-points, respectively.

Twenty-three sera were either antibody positive or doubtful on the first run using INGENASA. However, a re-run on all the positive sera (n = 5) using INGENASA and SVANOVIR ASFV-Ab ELISA tests, and all doubtful sera (n = 18) using SVANOVIR, resulted in none of the samples being positive for ASF antibodies with either of the two different ELISA tests used.

### Differential diagnosis

In total 239 samples were analyzed for presence of antibodies and nucleic acids specific to CSFV and PRRSV, respectively. All samples tested negative for CSF and PPRS in ELISA as well as in RT-PCR. This is also reported as preliminary results in a student thesis [13].

### Herd categories and risk factors

The numbers of farms according to size were 185 (78.7 %) and 50 (21.3 %) for small and medium-scale pig farms, respectively, with six missing values at first sampling. At second sampling, the numbers were 179 (76.5 %) and 49 (20.9 %) farms for the small-scale and medium-scale farms, respectively. Results from the questionnaire are presented in Tables 1 and 2. There were more farms with high proportions of improved breeds compared to those with local breeds of pigs. There were comparably more farms that borrowed boars from other farms for mating than those that did not, more farms sourcing replacement stock from neighbouring farms than those obtaining replacement stock generated on their own farms. For small-scale farms, those with none of the biosecurity measures (fences, controlled access to pens, foot-baths) were greater in number than those with at least one of the biosecurity measures in place (Table 1). The medium-scale farms had more farms that had at least one biosecurity measure than those that had none (Table 2). Feeding swill was common in both small and medium-scale farms.

**Table 1:** Risk factors for small-scale farms for ASF reports between first and second sampling (n = 179), 2010–2011

		^a^ ASF between			
Risk factor		No^b^ (%)	Yes^b^ (%)	NA^b^ (%)	Total (n)
Awareness ^c^	Not aware about ASF	67	0	33	3
	Aware about ASF	69	5	26	170
	NA ^d^	33	0	67	6
Biosecurity measures ^e^	At least one	70	3	28	40
	None	68	6	27	127
	NA	67	0	33	12
Borrow boar	No	59	0	41	27
	Yes	70	5	25	136
	NA	69	8	23	13
Breed	Local	67	4	28	67
	Improved	69	5	26	111
	NA	0	0	100	1
Duration of enterprises					
	Less or equal to 10 years	67	3	30	112
	Greater than 10 years	74	6	20	50
	NA	63	13	25	16
Ectoparasites control	No	74	0	26	27
	Yes	68	5	28	145
	NA	57	14	29	7
Feeding swill	No	72	9	19	47
	Yes	68	3	29	130
	NA	0	0	100	2
Labour	Family	69	4	26	160
	Hired	69	6	25	16
	NA	0	0	100	3
Pets present on farm	No	67	5	28	111
	Yes	68	4	28	57
	NA	82	0	18	11
Piglets housing	Piglets housing present	69	5	25	91
	Piglets not housed	71	2	27	83
	NA	0	20	80	5
Pigs housing	Pig housing present	66	5	29	111
	No pig housing	75	0	25	64
	NA	25	50	25	4
Replacement stock	Own farm	73	5	23	66
	From neighbouring farms	67	4	29	106
	NA	43	14	43	7
Wild pigs (bush pigs) contact	No	72	5	22	98
	Yes	56	11	33	9
	NA	64	3	33	72

^a^ ASF between- Reports of ASF on farms during the one year between the first and second sampling visits. This is the dependent variable and the row variables in the table are the independent variables

^b^ The numbers in each of the cells under columns No, Yes and NA are relative proportions (percentages) of the total number of pigs (column Total, n) in each of the table rows

^c^ Awareness encompasses those farms where farmers expressed having knowledge on the symptoms, spread, control and prevention measures for ASF

^d^ Missing values

^e^ Biosecurity measures considered were presence of a fence to the farm, controlled entrance to the pig pens (presence of gate/door) and presence of foot baths

**Table 2:** Risk factors for medium-scale farms for ASF reports between first and second sampling (n = 49), 2010–2011

		^a^ASF between			
		No^b^ (%)	Yes^b^ (%)	NA^b^ (%)	Total (n)
Awareness ^c^	Not aware about ASF	50	0	50	2
	Aware about ASF	85	4	11	46
	NA ^d^	100	0	0	1
Biosecurity measures ^e^	At least one	83	4	13	24
	None	85	5	10	20
	NA	80	0	20	5
Borrow boar	No	100	0	0	17
	Yes	78	4	19	27
	NA	60	20	20	5
Breed	Local	71	14	14	7
	Improved	85	2	12	41
	NA	100	0	0	1
Duration of enterprises					
	Less or equal to 10 years	85	3	12	34
	Greater than 10 years	75	8	17	12
	NA	100	0	0	3
Ectoparasites control	No	75	0	25	4
	Yes	86	2	12	43
	NA	50	50	0	2
Feeding swill	No	88	6	6	17
	Yes	81	3	16	32
	NA	0	0	100	1
Labour	Family	76	6	18	34
	Hired	100	0	0	9
	NA	100	0	0	6
Pets present on farm	No	84	4	12	25
	Yes	86	0	14	21
	NA	67	33	0	3
Piglets housing	Piglets housing present	80	5	15	40
	Piglets not housed	100	0	0	8
	NA	100	0	0	1
Pig housing	Pig housing present	82	5	14	44
	No pig housing	100	0	0	4
	NA	100	0	0	1
Replacement stock	Own farm	87	0	13	15
	From neighbouring farms	82	6	12	33
	NA	100	0	0	1
Wild pigs (bush pigs) contact	No	82	6	12	33
	Yes	100	0	0	1
	NA	87	0	13	15

^a^ ASF between- Reports of ASF on farms during the one year between the first and second sampling visits. This is the dependent variable and the row variables in the table are the independent variables

^b^ The numbers in each of the cells under columns No, Yes and NA are relative proportions (percentages) of the total number of pigs (column Total, n) in each of the table rows

^c^ Awareness as a variable encompasses those farms where farmers expressed having knowledge on the symptoms, spread, control and prevention measures for ASF

^d^ Missing values

^e^ Biosecurity measures considered were presence of a fence to the farm, controlled entrance to the pig pens (presence of gate/door) and presence of foot baths

The risk factor analysis of reported outbreaks of ASF during the study period did not produce any statistically significant predictors (Table 3).

**Table 3:** Univariable logistic regression model on pig farms for ASF reports between the samplings (n = 233), 2010–2011

		ASF between		
Independent variables		OR	95 % CI	*P*-value
Awareness ^a^	Not aware about ASF	1		
	Aware about ASF	-	-	-
Biosecurity measures ^b^	None	1		
	At least one	1.01	(0.26;3.37)	0.99
Borrow boar	No	1		
	Yes	-	-	-
Breed	Local	1		
	Improved	0.92	(0.28;3.57)	0.90
Duration of enterprise				
	Less or equal to 10 years	1		
	Greater than 10 years	2.28	(0.61;8.55)	0.21
Ectoparasites control	No	1		
	Yes	-	-	0.99
Farm size	Small-scale	1		
	Medium-scale	2.16	(0.61;7.15)	0.21
Feeding swill	No	1		
	Yes	0.66	(0.20;2.31)	0.49
Labour	Family	1		
	Hired	1.78	(0.38;6.45)	0.41
Pets present on farm	No	1		
	Yes	0.64	(0.14;2.33)	0.53
Piglets housing	Piglets housing present	1		
	Piglets not housed	1.87	(0.52;8.77)	0.37
Pig housing	Pig housing present	1		
	No pig housing	-	-	0.99
Replacement stock	From own stock	1		
	From neighboring farms	1.63	(0.45;7.64)	0.49
Wild pigs (bush pigs) contact	No	1		
	Yes	2.04	(0.10;14.19)	0.53

ASF between - Reports of ASF on farms during the one year between the first and second sampling visits

*OR* odds tatio, *CI* confidence interval, *ASF* African swine fever

- indicates that the model was inestimable because of skewed data

^a^Awareness as a variable encompasses those farms where farmers expressed having knowledge on the symptoms, spread, control and prevention measures for ASF

^b^Biosecurity measures considered were presence of a fence to the farm, controlled entrance to the pig pens (presence of gate/door) and presence of foot baths

## Discussion

ASF has had a global upsurge, and has been reported in at least 26 countries in SSA alone during the last few years [1]. The disease is considered endemic in domestic pig populations in many of these countries, but data on incidence rates is scarce. In our study population, more than 5 % of the farms reported incursions of ASF during the one-year study period (ASF between). Albeit based on farmer reports, this gives a rough estimate of the incidence rate of the disease in the population. Nine of the 13 affected farms were located in areas in which we confirmed ASF during this period (data not shown), supporting the accuracy of the reports. The estimated incidence rate for the period prior to the study (ASF prior) was numerically higher compared to ASF between, but the difference was not statistically significant. This latter estimate is likely to be less accurate than for ASF between, because it includes farmers’ perception of time since last experience of ASF and was therefore excluded from analysis of risk factors. Record keeping among smallholder pig farmers in the region is generally poor [14].

Important differential diagnoses to ASF such as CSF and PRRS, have never been reported in Uganda or in neighboring countries [15], and our study also failed to demonstrate presence of or exposure to these diseases in the study population. Moreover, during the period 2010–2012, we investigated around 50 reported outbreaks of suspected ASF in Uganda, including several in the study area, and in all but two ASF was confirmed, clearly suggesting ASF as the most prevalent cause of disease with high mortality in pigs in the region. All samples (n = 80) from four of these outbreaks, including the two in which ASF was not confirmed, were also tested for CSF and PRRS with negative results in all but one sample which was weakly positive on PRRS ELISA [13]. Given that only one out of a total of 319 samples tested positive for PRRS antibodies, the weakly positive result was interpreted as false positive. Our case definition was based on farmer reports of outbreaks of disease with clinical signs suggestive of, but not pathognomonic to ASF, which could imply a risk for misclassification. However, given the level of awareness of ASF demonstrated by the farmers, the very dramatic clinical signs typically associated with ASF, and the probable absence of the most important differential diagnoses in the study population, this risk is considered low.

Bacterial diseases such as erysipelas, which do occur in the study area, are also often mentioned as differential diagnoses to ASF, due to similar clinical signs in the individual animal. However, in contrast to ASF, erysipelas is a curable disease that most often affects individual animals rather than entire herds, and was therefore not considered in the study.

Several authors have reported high prevalences of ASF, based on detection of ASFV DNA in blood, serum and/or tissues using PCR in apparently healthy and often seronegative domestic pigs originating from locations from which no disease had been reported [9–11, 16]. Although the importance of this finding is debated [1], it has raised concern of a role of long-term carriers, which would not be detected through clinical or serological surveillance, in the maintenance of the disease in endemically infected populations. In this study, we did not find any evidence supporting a role of long-term carriers. No PCR positive animals were detected during the first sampling, only a few during the second, and no antibody positive animals during either of the samplings. The two farms with PCR positive animals were both located in areas that had recently reported ASF outbreaks, and in one of them, the farmer reported having had deaths on the farm as a result of ASF just prior to our visit. The three PCR positive animals were seronegative and it is likely that the absence of seroconversion reflected sampling at an early stage of infection before clinical signs had developed and an antibody response had been mounted. A neighbouring farm also reported having had ASF outbreak at the second sampling, which may suggest possible spread from either of the two farms given the management practices and risk factors mentioned earlier.

A number of studies have shown that pigs that survive ASF, may have persisting infection, with detectable virus only in lymphoid tissues, and not in blood or serum, up to 2–3 months after infection [17–19]. Moreover, it has been demonstrated that pigs that survive infection have detectable levels of antibodies that persist for at least 1–2 years, with a half-life estimated at 1.8 years [1, 19, 20]. A scenario with persistent infection only in lymphoid tissues in seronegative pigs has not been described to our knowledge. In this study, no pigs were sacrificed, and therefore lymphoid or other tissues could not be tested, but because no antibody positive pigs were found, we do not believe this has affected our results.

To confirm that any positive animals found during the first sampling were persistently positive, as would be expected from long-term carriers, our ambition was to resample the same animals during the second sampling round. This was in many cases not possible. However, given that no animals were positive, neither on PCR nor on ELISA, during the first sampling round, and only three were PCR positive during the second (all closely linked with outbreaks directly affecting study farms), this did not affect the interpretation of our results nor the conclusion regarding presence or absence of potential long-term carriers in the study population.

The absence of detectable seropositivity in the study population is in accordance with several studies suggesting a very low seroprevalence of ASF in domestic pig populations in eastern Africa [21, 22]. The low seroprevalence, in spite of a relatively high incidence of ASF, reflects circulation of a highly virulent strain of ASFV with high mortality, but is likely also a result of the common practice of selling off pigs for slaughter as soon as an outbreak of ASF occurs, to salvage some income from the dying or in-contact pigs [1, 22]. A recent publication from Uganda, however, presents results from a combined slaughterhouse and on-farm study with sampling of apparently healthy pigs and reports a seroprevalence of above 50 %, indicating circulation of low virulent viruses and possibly development of natural resistance [12]. This is in vast contrast not only to our results, but also to those from the several other published studies from the region [10, 16, 21, 22]. The serological analyses in the aforementioned study [12], however, were performed using an in-house ELISA based on the semipurified ASFV antigen. This method is known to give a certain proportion of false positive test results, especially with poorly preserved samples as is often the case under African conditions due to the hot climate and not always functioning cold chain. Therefore, unexpected positive results, such as in this case, should always be confirmed using an alternative test before conclusions can be drawn from the results [23, 24].

The vast majority of pig farms included in the study were small-scale (78.7 %) with a maximum of ten pigs while medium-scale farms (11–200 pigs) accounted for only 21.3 %. There were high proportions of farms with pigs and piglets that were not housed. There were differences in the relative importance of risk factors and the extent of use of biosecurity measures between medium-scale and small-scale farms (Tables 1 and 2). This difference in distribution of ASF risk factors and biosecurity measures between the two categories of farms agrees with the findings reported by Costard et al [25] in an earlier study in Madagascar. On both categories of small and medium-scale farms, farmers reported improper disposal of carcasses which included selling of dead/dying pigs for slaughter, throwing carcasses in bushes and giving pork from diseased pigs to neighbours (data not shown). These practices will certainly promote the spread of ASF through movement of the infected pigs, contaminated carcasses and pork products especially during ASF outbreaks. None of the risk factors were, however, significantly associated with ASF outbreaks between the two visits, probably partly due to low power of the study, imprecise identifications of the risk factors, and to some extent missing values (NA). However, the proportion of missing values was usually low (below 10 %), except for the question on contacts with wild pigs, and we believe that the missing answers would be non-differential thus only leading to a bias towards the null hypothesis.

## Conclusions

Our results indicate a high incidence rate of ASF in the study area, and demonstrate that long-term carriers are not needed to explain the maintenance of the disease in the population. Potential ASF risk factors were present on both small and medium-scale pig farms, although small-scale farms exhibited a higher proportion with multiple potential risk factors and lacking any implementation of biosecurity measures. However, no risk factors were significantly associated with ASF reports during the study.

## Methods

### Study area and farm selection

A longitudinal study was carried out in the districts of Masaka and Rakai in central Uganda (Fig. 1). A total of 24 sub-counties were selected by targeting sub-counties with the largest number of pig farms as indicated by the district veterinary officers. A sampling frame of villages within selected sub-counties was generated, and five villages were randomly selected per sub-county. Two farmers within each of these villages were selected in consultation with the district veterinary officers. In some cases, the veterinary officers suggested new villages as replacements based on unavailability of pigs in some of the originally selected villages. A farm was only included in the sample if the farmer affirmed that he/she planned to keep pigs for the next one year and if the farm reared at least three pigs. The farms were visited twice, during June to October 2010 and during March to June 2011. All farms were geo-referenced (Fig. 1).

### Data collection

Information was collected using a questionnaire with closed and open-ended questions, after getting informed consent. The questionnaire was initially discussed with the local veterinary personnel to make sure they all understood the questions similarly since they had to be translated into Luganda, the local language. The enumerators made sure that the questions were understood by the respondents. During the first visit, farmer awareness and knowledge of ASF was investigated. Also, the farmers were asked whether their pigs had had any incidences of infectious disease with clinical signs suggestive of ASF (that is mortality, fever, loss of appetite, reddened skin) on their farm in the previous 1–2 years. In addition, information on management practices, biosecurity measures and general information on the farms were taken (Tables 1 and 2). During the second visit, the farmers were asked specifically if they had had outbreaks of ASF since the previous visit and also to describe the outcome of these outbreaks in terms of clinical signs and mortality.

### Sample collection

Permission was sought to sample three pigs from farms where interviews were conducted. Three pigs were chosen from each herd, restrained by the muzzle using a commercially available pig catcher and examined by a veterinarian. All the pigs sampled were at least three months of age. Sampling was done twice on the pig farms (*i.e*. at each visit at time periods as indicated above), with an ambition of including the same pigs in the two samplings. This, however, was in many cases not possible, because pigs had been sold or had died or the farmers could not be found at the time of the second sampling. Whole blood was taken from the jugular vein into appropriate vacutainers. Serum samples were similarly collected from each pig into appropriate serum vacutainers (BD, New Jersey). Whole blood was aliquoted into duplicate 2 ml cryovials (Cryo.s, Greiner Bio-one, Wemmel). Serum tubes were centrifuged at 2000 g for 10 mins to separate serum from clotted blood serum aliquoted into duplicate 2 ml cryovials. The aliquoting and centrifugation were done at the regional district laboratories every evening after farm visits. The duplicate cryovials were later transported on ice in cool boxes to the Molecular Genetics Laboratory in the College of Agriculture and Environmental Sciences, Makerere University for storage at -20 **°**C and -80 **°**C as working and long-term storage sample aliquots, respectively.

All handling of animals including sampling was carried out, or overseen, by District Veterinary office staff in accordance with their national mandate. The district veterinary office, under the Ministry of Agriculture Animal Industry and Fisheries (MAAIF) has the official mandate to carry out investigations related to animal disease in the country.

### ASFV DNA detection

In order to check for the presence of ASFV nucleic acids, the samples were prepared for total DNA extraction. One hundred microlitres of anticoagulated blood from each pig sample for the three pigs per farm was collectively pooled and thoroughly mixed to make 300 μl. From this pool, 100 μl was used for total genomic DNA extraction using the DNeasy Blood & Tissue kit (Qiagen, Duesseldorf) following the manufacturer’s protocol. In all extraction steps, a negative control was included. The extracted DNA was either immediately used in the RT-PCR assay, or stored at -20 °C until used. For the detection of ASFV DNA, a commercially available ASF RT-PCR Tetracore® assay (Tetracore Inc., Rockville, Maryland) was used according to the instructions of the manufacturer. The assay was optimized for use on a SmartCycler® (Cepheid Inc., Sunnyvale, California), a 10 kg portable instrument that is operated by a laptop computer [26].

For the pooled samples that tested positive, the entire procedure from DNA extraction to RT-PCR was repeated for individual pig blood samples to identify which of the three pigs was actually positive.

### ASFV antibody detection

For the detection of antibodies against ASFV, a commercially available blocking ELISA (INGEZIM PPA Compac 11.PPA.K3, INGENASA, Spain), recommended by the OIE, was used in accordance with instructions from the manufacturer. It targets the VP73 viral protein and is reported to have a sensitivity and specificity of 95–98 % [27]. The positive and doubtful samples were re-tested for confirmation using the same INGENASA ELISA and the recently released SVANOVIR® ASFV-Ab (Boehringer Ingelheim Svanova, Uppsala, Sweden) indirect ELISA. The SVANOVIR® ASFV-Ab ELISA kit (screening plates) was used according to the instructions by the manufacturer.

### Differential diagnosis

To reduce the risk of misclassification, a subset of the samples from the first sampling round was also tested for classical swine fever, the most important differential diagnosis to ASF. In addition, the same subset was tested for presence of porcine reproductive and respiratory syndrome (PRRS) virus, which in its most virulent form can cause a disease with clinical signs resembling ASF. For antibody detection, commercial ELISA kits were used (CSF Ab test and PRRS X3 antibody test, IDEXX Laboratories Inc., Maine, USA). For virus detection, commercially available CSF and PRRS RT-PCR kits were used (Tetracore Inc., Rockville, Maryland). All tests were run according to the instructions by the manufacturers.

### Statistical analysis

Incidence rates for ASF in the study population were estimated based on the farmer reports of outbreaks 1–2 years prior to first sampling (ASF prior; 1.5 years taken as denominator) and of outbreaks between first and second sampling (ASF between; 1 year as denominator), respectively. A case was defined as a herd with a reported outbreak during the period of interest of an infectious disease with clinical signs suggestive of ASF, i.e. high mortality, high fever and loss of appetite, with or without discoloration of the skin, diarrhea, abortions etc.

A test of whether the two incidence estimates were significantly different was computed in R. Logistic regression models were used to assess the association between reports of outbreaks during the period of interest of an infectious disease with clinical signs suggestive of ASF (response variable) and management practices (independent variables). In this analysis, only outbreaks that occurred between first and second sampling were considered. None of the risk factors were associated with the response variable (*P* >0.25) and no multivariable model was therefore attempted. Data was analysed using R statistical package (Version 2.15.2) for logistic regressions [28]. In this study, the farms were grouped into two categories basing on the number of pigs reared. They included; small-scale (1–10 pigs) and medium-scale pig farms (11–200 pigs).

Positive and negative test results based on RT-PCR and ELISA were entered into a Microsoft Excel spreadsheet (Microsoft Corporation, Redmond, Washington). Apparent and true prevalence were computed using epiR package in R (Version 2.15.3) and as earlier described [29] using sensitivity and specificity of 90 and 100 % respectively [26].

## Abbreviations

ASF: African swine fever
ASFV: African swine fever virus
CSF: Classical swine fever
Ct: Cycle threshold
DNA: Deoxyribonucleic acid
ELISA: Enzyme-linked immunosorbent assay
PCR: Polymerase chain reaction
PRRS: Porcine reproductive and respiratory syndrome
RT-PCR: Real time polymerase chain reaction
